# Cognitive Impairments Preceding and Outlasting Autoimmune Limbic Encephalitis

**DOI:** 10.1155/2016/7247235

**Published:** 2016-01-10

**Authors:** Robert Gross, Jennifer Davis, Julie Roth, Henry Querfurth

**Affiliations:** ^1^Department of Neurology, Icahn School of Medicine at Mount Sinai, New York, NY 10029, USA; ^2^Department of Psychiatry, Rhode Island Hospital, Brown University School of Medicine, Providence, RI 02903, USA; ^3^Department of Neurology, Rhode Island Hospital, Brown University School of Medicine, Providence, RI 02903, USA

## Abstract

Mild cognitive impairment (MCI) can be the initial manifestation of autoimmune limbic encephalitis (ALE), a disorder that at times presents a diagnostic challenge. In addition to memory impairment, clinical features that might suggest this disorder include personality changes, agitation, insomnia, alterations of consciousness, and seizures. Once recognized, ALE typically responds to treatment with immune therapies, but long-term cognitive deficits may remain. We report two cases of patients with MCI who were ultimately diagnosed with ALE with antibodies against the voltage gated potassium channel complex. Months after apparent resolution of their encephalitides, both underwent neuropsychological testing, which demonstrated persistent cognitive deficits, primarily in the domains of memory and executive function, for cases 1 and 2, respectively. A brief review of the literature is included.

## 1. Introduction

Mild cognitive impairment (MCI) has a prevalence of 16–20% in over 65 population [1–3]. 6% constitute amnestic MCI with a high likelihood to advance to Alzheimer's disease [2]. MCI is a heterogeneous entity and includes some patients on an indolent path to a nonneurodegenerative and potentially treatable encephalopathy, as well as those with lingering deficits from such a disease process presenting in the recovery phase. If the illness evolves rapidly, a variant of AD may still be considered; however, the presence of atypical signs should raise suspicion for an infectious, paraneoplastic, or autoimmune etiology. 51% of ALE patients may be seronegative [4].

Here we report two cases of antivoltage gated potassium channel complex (VGKCC) encephalitis that initially presented as MCI with atypical features. Both had early nonconvulsive spells ascribed to limbic or dystonic seizures. After remission, both were left with MCI corresponding to amnestic or dysexecutive syndromes.

## 2. Case 1

A 71-year-old woman first presented to us for progressive memory complaints over one year in the context of the loss of a loved one and stress in the family. Past medical history included anxiety, lumbago, psoriasis, and multiple drug sensitivities including to a steroid injection. She had a high school education and worked as a telecommunication operator until retiring at age 65. Initially she would become confused with directions, missed appointments, and had occasional word finding difficulty but then had increasing problems with calculations and penmanship. She had forgotten her daughter's recent pregnancy. She began to have infrequent incidents consisting of rising paresthesiae, during which she was seen to tense her body, clench her teeth, become flushed, and look afraid. They resembled “panic attacks.” These events were associated with amnesia for the event and were followed by confusion. She had also been having myoclonic jerks, though these had subsided by the time of her initial presentation. A first neurologist started her on levetiracetam for suspected seizures, but this was discontinued after causing delirium. A second neurologist recorded a Mini Mental Status Score (MMSE) of 29/30, a Montreal Cognitive Assessment (MOCA) of 24 (1/5 verbal recall), diagnosed depression/anxiety, and initiated donepezil. This too was discontinued soon thereafter for ineffectiveness.

On our initial evaluation, her Clinical Dementia Rating (CDR) was 0.5 and MMSE was 28 (1/3 delayed verbal recall). Formal neuropsychological testing showed a largely intact cognitive profile with the exception of inefficient learning and impaired recall of verbal information. The Dementia Rating Scale (DRS) score was 139. The Beck Inventory was 8, not suggestive of active depression. MRI brain showed moderate global cerebral atrophy, though not specifically in the mesial temporal lobes, and small vessel ischemic changes. Fluorodeoxyglucose positron emission tomography (FDG PET) of the brain showed no areas of abnormal metabolism. She was diagnosed with MCI, amnestic type, and started on galantamine. There was clinical stabilization.

A little over a year from her presentation, she developed increasing anxiety, new auditory hallucinations, sleep disturbance, compulsive behaviors, and attacks resembling panic. Escitalopram and alprazolam were prescribed, subsequently changed to sertraline and lorazepam, and quetiapine was added. Memory complaints resurfaced: an MMSE was 27 (0/3 recall). Galantamine was discontinued due to diarrhea, cramps, and weight loss, and she was started on rivastigmine, to which memantine was added.

**Figure 1:**
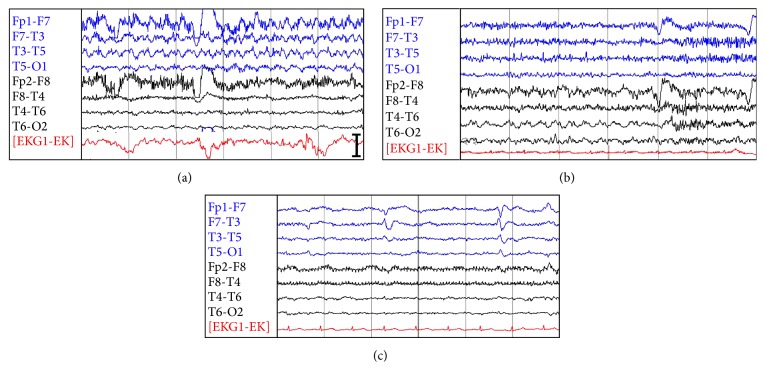
Sample 24 hr video EEG tracings from case 1. Push button activation processes and video recorded panic/fear attacks corresponding to left (a) alternating with right (b) temporal lobe activations and electrographic seizures. (c) The interictal record showing periodic left anterior sharp wave epileptiform discharges. 1 Hz time intervals, 2 *µ*V/mm sensitivity, and bar 100 *µ*V.

Over the next 3 months increasing anxiety led to a brief psychiatric evaluation in the ED. Mild hyponatremia (131 meq/L) was noted. A routine electroencephalogram (EEG) around this time showed bitemporal sharp waves. By 18 months after presentation, mental status had sharply deteriorated with a larger number of stereotyped panic-fear attacks. 48-hour ambulatory EEG showed interictal periodic sharp and slow wave discharges in bilateral temporal lobes, and bitemporal electrographic seizures correlated to these clinical events, hereafter referred to as seizures (Figure 1). She was promptly started on phenytoin and lamotrigine and admitted to the hospital.

MMSE during this hospitalization was 15/30. Noncontrast head CT showed global atrophy but no acute findings. Cerebrospinal fluid (CSF) showed 1 nucleated cell, protein 32 mg/dL, glucose 63 mg/dL, and negative infectious studies. CSF 14-3-3 was mildly elevated at 2.6 ng/mL (normal < 1.5). Cytology was negative. CSF A*β*_(1–42)_ was 148 pg/mL (reduced), and CSF total tau was 223 pg/mL (elevated) for an A*β*42-tau index of 0.29, but CSF phospho-tau (44 pg/mL) was not elevated, an inconclusive pattern. Calcium and thyroid stimulating hormone (TSH) were normal. Send-out testing subsequently returned borderline elevated for anti-VGKCC titers (498 pmol/L) and strongly positive for anti-leucine-rich glioma inactivated 1 protein (LGI1) antibodies by immunocytochemistry. She was diagnosed with LGI1 LE and readmitted for a 5-day course of intravenous immunoglobulin (IVIg).

After a 2-3-week period of recovery (MMSE = 27), she relapsed and was readmitted for wandering, emotional lability, agitation, and suicidal ideation. A second course of IVIg was supplemented with 4 days of high dose IV methylprednisolone. Shortly after finishing, she began to show signs of improvement. Epileptic fits stopped, and agitation and psychosis subsided. MMSE improved to 28, but insight remained impaired, and labile behavior persisted. 24 hr EEG revealed no further evidence of epileptiform activity. In rehabilitation, lamotrigine was discontinued due to rash, and she was discharged home on quetiapine, phenytoin, and low-dose lorazepam.

**Figure 2:**
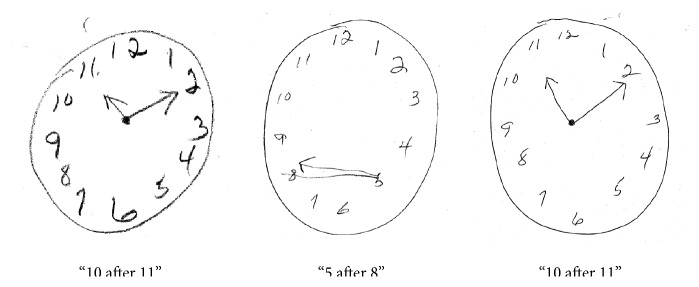
From left to right, clock drawing in 13 months, 32 months, and 38 months from symptom onset.

Repeat neuropsychological testing 1 month after discharge, compared to her previous baseline, demonstrated declines in working memory, psychomotor speed and executive function, and persistently impaired verbal memory. By 24 months after presentation, a third neuropsychological evaluation showed that while many of these changes had reversed, she continued to have difficulty in verbal memory and visuospatial skills (Table 1), albeit normalization of clock draw (Figure 2).

## 3. Case 2

A 72-year-old man first presented to us for evaluation of a one-month history of abnormal movements of his limbs and face associated with alteration of awareness and subsequent amnesia for the events. Past medical history was notable for recurrent bronchitis, dyslipidemia, abdominal and thoracic aortic aneurysms, and essential tremor. He had been on oral steroids intermittently over several years for bronchitis and was on a tapering schedule when the abnormal movements began. They consisted of stereotyped myoclonic jerking and dystonic posturing of the right more than left upper extremity and face. He exhibited hand clenching, index finger or thumb extension, shoulder elevation, and facial contortion. The attacks occurred several times a day, lasting from a few seconds up to a minute each, and were becoming more frequent, occurring every 15–20 minutes and at night. During them, he would be less responsive and thereafter lethargic. He occasionally expressed the delusion of the TV transmitting commands to his wrist. His family reported behavioral changes over the previous month: his personality became more subdued. They also noticed short term memory and word finding difficulties. He had no previous history of seizures or mood disorders. The neurological and medical reviews of symptoms were unrevealing.

He had presented to a different hospital 2 weeks earlier and had undergone evaluation, including an MRI brain (1.5 T) with gadolinium, MRA head and neck, and routine EEG, all unremarkable. A neurologist diagnosed partial motor seizures and treated him with valproic acid at first, sequentially adding levetiracetam and zonisamide, with little benefit. Another neurologist obtained a Kokmen mental status screen score of 36/38. He was then admitted to university hospital for 48-hour video EEG (VEEG).

On initial examination, he was alert and oriented to place and date and followed commands. Speech and language were normal. He endorsed ideas of reference but was not overtly psychotic. His affect was flattened and he was mildly sluggish but was able to read and name the months backwards. Word recall was 1-2/3 at 10 minutes. There was bilateral postural tremor, but the remainder of the elemental neurological exam was normal.

**Figure 3:**
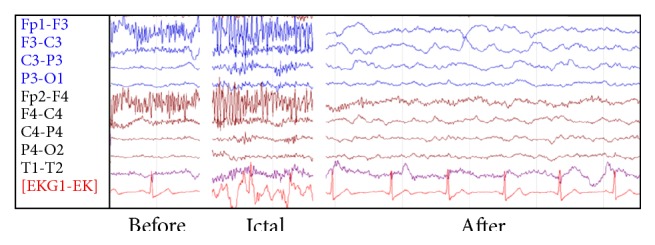
Sample tracing from 24 hr VEEG of case 2. Push button activation and video recorded faciobrachial dystonic seizures; preictal, ictal (largely muscle artifact), and postictal recording samples, the latter displaying left greater than right side slowing in the theta and delta range, thereafter becoming symmetric (not shown) (bar 100 *μ*V).

Over the first 48 hours on VEEG, numerous brief, right greater than left stereotyped episodes were captured, all without associated distinct epileptiform discharges, though many had an electrographic correlate consisting of left temporal bursts of midtheta rhythms seen immediately following the push button events (Figure 3). Valproic acid and zonisamide were discontinued, and phenytoin was added. Lumbar puncture was traumatic and had 1053 RBCs and 9 nucleated cells in the first tube, 42 RBCs and 5 nucleated cells in the fourth tube, protein 50 mg/dL, and glucose 69 mg/dL. He was started empirically on acyclovir, which was stopped after 3 doses when PCR for HSV 1 and HSV 2 returned negative.

Hospital course was complicated by acute kidney injury and pulmonary edema with respiratory failure resulting in brief intubation and stay in the Medical ICU. Several days after extubation and transfer back to the ward, he was noted to have impairments in insight, problem solving, attention to multistep commands, and short term memory. He became labile and impulsive and went through a brief period of threatening visual hallucinosis. Encephalopathy workup revealed mildly elevated titers of anti-thyroid peroxidase (TPO) and anti-thyroglobulin (TG) antibodies (137.8 IU/mL and 73.1 IU/mL, resp.); he was tentatively diagnosed with Hashimoto encephalopathy and treated with IV methylprednisolone for 5 days. The movement spells grew less frequent, were of shorter duration, and appeared less dramatic with the addition of clonazepam and then haloperidol to his regimen.

MRI brain (3T) with gadolinium was again unremarkable. An extensive infectious workup was similarly unrevealing, including for tickborne and viral pathogens. CSF 14-3-3 was negative. Serum ceruloplasmin and TSH were normal. Urine delta ALA and serum porphobilinogen were normal. Hospital course was further complicated by mild hyponatremia (130 meq/L) and a small retroperitoneal bleed that was conservatively managed. He was discharged home after a 10-day stay on phenytoin 150 mg twice daily and low doses of clonazepam, haloperidol, and prednisone.

After discharge, autoimmune encephalitis panel returned with elevated titers of anti-VGKCC autoantibodies at 572 pmol/L. Other autoantibodies (Hu, Ma1, CV2, Amphiphysin, NMDA, and GAD65) were absent (specific anti-LGI1 testing was not yet available). He underwent a workup for underlying malignancy with CT chest, abdomen, and pelvis, which was negative. Haloperidol was discontinued. His former personality had returned, and he remained seizure-free at last follow-up on low doses of phenytoin and prednisone. Neuropsychological testing 2 years after discharge revealed lasting impairments in executive function (divided attention and fluency) and low-average psychomotor speed. Notably, memory testing proved normal (Table 1).

## 4. Discussion

These two cases share several similar features. Both patients developed insidious memory loss and behavioral changes over months qualifying initially as MCI or cognitive impairment/decline, no dementia (CIND). Complex partial and dystonic seizures occurred in case 1 and case 2, respectively. Furthermore, both developed hyponatremia. At the peak of their illnesses, both became confused and had hallucinations. Both underwent extensive workups before the correct diagnosis was revealed. Both had variable responses to symptomatic treatments, including anticonvulsants, but improved dramatically with immunotherapies. Finally, there were persistent cognitive deficits in both patients long after apparent resolution of their ALE.

Clinically, VGKCC LE overlaps with other ALE cases. Case 2 highlights the fact that steroid-responsive encephalopathy associated with autoimmune thyroiditis (SREAT or Hashimoto's encephalopathy [HE]) is among the top differential diagnoses to consider. Confusion, myoclonus, hallucinations, psychosis, and seizures are found in both [5–7], though hyponatremia is commonly seen with VGKCC LE, while stroke-like events, such as episodes of transient aphasia, frequently occur in HE [8]. Anti-TPO and anti-TG antibodies are often present in cases of VGKCC LE and are found in 10–15% of the general population [9], so their presence should be considered nonspecific. Neuroimaging and CSF evaluations are not sensitive: MRI shows high signal in the medial temporal lobes in about half to 80% cases of VGKCC LE, and CSF is often normal [6, 7, 9–12].

Serum autoantibodies against the VGKCC have been associated with acquired neuromyotonia (Isaac's syndrome), Morvan syndrome, epilepsy, and ALE [11]. Most autoantibodies are not directed against the VGKC subunits Kv1.1, 1.2, and 1.6, but rather against associated proteins LGI1 and contactin-associated protein-2 (CASPR2). LGI1 is the most common target of pathogenic autoantibodies in VGKCC LE [6, 12]. A neuronal protein associated with Kv1.1 in presynaptic terminals of the central nervous system, LGI1, is secreted and serves as a cell surface ligand for the ADAM22 and ADAM23 transmembrane proteins. It is strongly expressed in the hippocampus; autopsies of LGI1 LE have demonstrated CD45 and CD8+ T cell lymphocytic and CD68+ microglial infiltrates and hippocampal neuronal loss, the latter predominantly in the CA4 layer [13, 14]. Genetic mutations in* LGI1* are seen in autosomal dominant lateral temporal epilepsy [15] and in autosomal dominant partial epilepsy with auditory features [16]. It has been demonstrated that autoantibodies to LGI1 disrupt the ligand-receptor interaction of LGI1 with ADAM22 or ADAM23, which leads to reduction in synaptic AMPA receptors [17].

The second case featured faciobrachial dystonic seizures (FBDS), which is highly correlated with LGI1 LE [10, 16]. These consist of brief dystonic movements of the face and ipsilateral arm and leg, usually involving alteration of consciousness, sometimes alternating sides, and occurring up to 360 times per day. The EEG may show frank ictal epileptiform potentials or, as with case 2, a suggestive electrographic correlate, or it may be unrevealing. FDG PET studies have demonstrated altered glucose metabolism in the temporal lobes and basal ganglia of patients with FBDS [18, 19].

There is no distinctive neuropsychological profile in VGKCC LE. While subacute cognitive impairment is the most common manifestation, with severe short term memory loss found in most cases, verbal or visual memory may be spared in a small subset [20]. A cohort of 19 patients with LGI1 LE underwent neuropsychological testing both at the height of the illness and at posttreatment follow-up (median interval between testing 254 days) [21]. The investigators found broad, profound cognitive impairment during the acute phase of illness and functional recovery in many cognitive domains after treatment, though anterograde amnesia persisted in about one-third of their patients. Notably, executive function tended to improve into normal ranges after recovery. These results are in accord with what we observed in our first patient. However, a nonamnestic, dysexecutive type MCI was the outcome of the other, perhaps reflecting more lasting basal ganglia-frontal dysfunction in patients with FBDS presentations. Our cases may be contrasted with another ALE syndrome that associated with AMPA-R antibodies. Those cases present more acutely with either frank confusion or fulminant encephalitis or as an isolated anterograde amnesia or mesiotemporal seizure but not apparently MCI [22].

Treatment of LGI1 LE consists of immunotherapies: steroids, plasmapheresis, and IVIg. There are no randomized controlled trials demonstrating superiority of one approach over another, though, in case series, patients tend to respond well to immune treatments and poorly to anticonvulsants [6, 7, 9, 12], again, similar to what we observed. Relapses can occur infrequently, especially as steroids are being titrated down [6], as case 2 experienced.

These cases underscore the difficulty associated with diagnosing LGI1 LE and confirm earlier findings that cognitive deficits may persist months to years after treatment [20]. When evaluating MCI, one should especially be vigilant for concomitant neuropsychiatric or epileptic disorders.

## Figures and Tables

**Table 1:** Summary of posttreatment neuropsychological testing of both patients. Case 1 is 6 months and case 2 is 2 years following last hospitalization.

Test	Case 1		Case 2	
	Raw score	Normed score	Raw score	Normed score
Global Cognition				
MMSE	30	Intact	30	Intact
Dementia Rating Scale	133	*T* = 43	139	*T* = 53
Attention				
Coding	56	*T* = 53	51	*T* = 50
Trails A (seconds)	27′′	*T* = 59	40′′	*T* = 41
Executive functions				
Trails B (seconds)	92′′	*T* = 49	**136**′′	**T** = 35
FAS	45	*T* = 58	**26**	**T** = 37
Animals	12	*T* = 40	20	*T* = 54
Rey Figure (organization)	5	*T* = 50	5	*T* = 45
Language				
Boston Naming Test	50	*T* = 47	60	*T* = 63
Visuospatial skills				
Rey Copy	**13**	**T** = 35	18	**T** = 60
Judgment of Line Orientation	11	*T* = 47	13	*T* = 55
Memory				
Verbal memory (HVLT-R)				
Total learning trials 1–3	5, 6, 7	*T* = 40	10, 10, 11	*T* = 65
Delayed recall	**0**	**T** = 19	**11**	**T** = 60
Recognition (Hits-FP)	9	*T* = 43	11	*T* = 54
Visual memory (BVMT-R)				
Total learning trials 1–3	3, 4, 11	*T* = 45	2, 8, 7	*T* = 43
Delayed recall	9	*T* = 55	8	*T* = 50
Recognition (Hits-FP)	5	>16th percentile	5	>16th percentile
Mood				
Beck Depression Inventory	1	WNL	12	WNL
